# Host genetics drives differences in cecal microbiota composition and immune traits of laying hens raised in the same environment

**DOI:** 10.1016/j.psj.2024.103609

**Published:** 2024-03-06

**Authors:** Alexandre Lecoeur, Fany Blanc, David Gourichon, Nicolas Bruneau, Thierry Burlot, Marie-Hélène Pinard-van der Laan, Fanny Calenge

**Affiliations:** ⁎ Université Paris-Saclay, INRAE, AgroParisTech, Jouy-en-Josas 78350, France; † INRAE, PEAT, Nouzilly 37380, France; ‡ NOVOGEN, Plédran 22960, France

**Keywords:** genetic, gallus gallus, gut microbiota, vaccine response, immunity

## Abstract

Vaccination is one of the most effective strategies for preventing infectious diseases but individual vaccine responses are highly heterogeneous. Host genetics and gut microbiota composition are 2 likely drivers of this heterogeneity. We studied 94 animals belonging to 4 lines of laying hens: a White Leghorn experimental line genetically selected for a high antibody response against the Newcastle Disease Virus (**NDV**) vaccine (**ND3**) and its unselected control line (**CTR**), and 2 commercial lines (White Leghorn [**LEG**] and Rhode Island Red [**RIR**]). Animals were reared in the same conditions from hatching to 42 d of age, and animals from different genetic lines were mixed. Animals were vaccinated at 22 d of age and their humoral vaccine response against NDV was assessed by hemagglutination inhibition assay and ELISA from blood samples collected at 15, 19, and 21 d after vaccination. The immune parameters studied were the 3 immunoglobulins subtypes A, M, and Y and the blood cell composition was assessed by flow cytometry. The composition of the cecal microbiota was assessed at the end of the experiment by analyzing amplified 16S rRNA gene sequences to obtain amplicon sequence variants (**ASV**). The 4 lines showed significantly different levels of NDV vaccine response at the 3 measured points, with, logically, a higher response of the genetically selected ND3 line, and intermediate and low responses for the unselected CTR control line and for the 2 commercial lines, respectively. The ND3 line displayed also a higher proportion of immunoglobulins (IgA, IgM, and IgY). The RIR line showed the most different blood cell composition. The 4 lines showed significantly different microbiota characteristics: composition, abundances at all taxonomic levels, and correlations between genera and vaccine response. The tested genetic lines differ for immune parameters and gut microbiota composition and functions. These phenotypic differences can be attributed to genetic differences between lines. Causal relationships between both types of parameters are discussed and will be investigated in further studies.

## INTRODUCTION

The immunocompetence of animals can be defined as the ability of the organism to generate a normal immune response after exposure to an antigen (Owens et al., 1999; Hine et al., 2014). It is therefore made up of different aspects, from the level of certain immune cells or molecules such as immunoglobulins (Kramer et al., 2003), the composition and quantity of certain cells required for an immune response (such as B and T cells in the blood) (Fairbrother and O'Loughlin, 1990; Maxwell and Robertson, 1998), or even the vaccine response that the organism can produce. Besides being one of the components of immunocompetence, vaccination is one of the most common and efficient ways of preventing economic losses resulting from pathogen infections and maintaining animal health (Marangon and Busani, 2007). Reinforcement of vaccination, relying for instance on a more frequent use of vaccination in addition to other disease management strategies, can also compensate for the reduction of antibiotics due to new regulations or strategic rearing choices. However, given the diversity of geographical regions, production types, and chicken species and breeds affecting the variability of vaccine response and infection outcomes (Pinard-van der Laan, 2002; Song et al., 2022), vaccines remain suboptimal for many endemic chicken diseases. Host genetic variability and variations in gut microbiota composition are 2 important drivers of this variation in efficacy, which can be measured using different parameters such as the quantity of antibodies at peak vaccination or the persistence of vaccination (Zimmermann and Curtis, 2018; Ciabattini et al., 2019).

Genetic selection could be an innovative way of improving vaccination efficacy, and more generally immunocompetence of poultry animals. Improving vaccine response of birds in farms may be achieved by tailoring vaccines to the host's genetics or by selecting animals with a superior immune response to vaccines. The optimal approach would be to synergize genetic improvement and vaccine adjustment (Gavora and Spencer, 1983). We have demonstrated the role of host genetics in vaccine response variation by successfully performing genetic selection of White Leghorn laying hens for a high vaccine response against Newcastle disease virus (**NDV**) (Pinard-van der Laan, 2002); the line which has been selected for over 25 years for a high antibody response against NDV vaccine 3 weeks after vaccination named ND3 (Pinard-van der Laan, 2002), and an unselected line named CTR (Zerjal et al., 2021). The ND3 line has a stronger humoral immune response, i.e. it produces between 2 and 4 times more antibody titer against NDV that the CTR line produces (Zerjal et al., 2021). More generally, several studies confirm the impact of host genetics on various other immune parameters (Heller et al., 1992; Parmentier et al., 2004; Berghof et al., 2018), and among them only a few focus specifically on vaccine responses. Several studies report the divergent selection of chickens based on the quantification of immune parameters, thus showing the existence of a genetic control of immune parameter variations. For example, we selected 2 other lines for high cell-mediated immune response and high phagocytic activity (Pinard-van der Laan, 2002). Lines diverging for their humoral responses to sheep red blood cells injection have also been successfully selected (Boa-Amponsem et al., 2001). Other studies estimated genetic parameters (heritabilities and genetic correlations) for immune parameters such as antibody response to *Pasteurella multocida* and *Mycoplasma gallisepticum*, T-cell-mediated response and phagocytosis in laying hens (Cheng et al., 1991), or heterophil:lymphocyte ratio in other avian species like turkey (Bayyari et al., 1997). Furthermore, the implication of the MHC locus in immunization (Zhang et al., 2022) and disease resistance is well documented (Kaufman, 2016; Pinard-van der Laan et al., 2020). In addition to the MHC locus, quantitative genetics studies show that several genetic loci are involved in the variations of specific immune parameters, for example, (Walugembe et al., 2020) found moderate to high estimates of heritability for all NDV response traits and identified potential candidate genes for postinfection growth rate and viral load. Consequently, genetic selection for immune parameters, including vaccine response, is theoretically feasible in an industrial breeding context.

It is now well established that host genetic variations have a significant influence on the gut microbiota composition in human as well as in livestock and domestic animal species. This has been well demonstrated for humans (Goodrich et al., 2014, 2017; Nichols and Davenport, 2021) and is increasingly studied in particular with the aim of improving health care (Tremaroli and Bäckhed, 2012). In pigs (Camarinha-Silva et al., 2017) for instance, have studied the effect of host genetics (heritability) on the composition of the intestinal microbiota, and vice versa (microbiability, which is defined as the phenotypic variance explained by gut microbial variance (Camarinha-Silva et al., 2017; Difford et al., 2018). The involvement of genetic variations in microbiota composition has also been demonstrated in broilers and in layers. Many studies identified differences in microbiota composition between distinct chicken lines. For instance, differences were identified between 2 lines (Green-legged Partridge vs. Sasso line C44) (Sztandarski et al., 2022), between 2 genetically different specific pathogen free lines (RIR vs. VALO chickens) (Chrzastek et al., 2021), between distinct inbred lines (Chintoan-Uta et al., 2020; Cazals et al., 2022). Other studies identified differences in microbiota composition between lines divergently selected for particular traits such as adiposity characteristics (Hou et al., 2016), feather pecking behavior (van der Eijk et al., 2019) or digestibility (Borey et al., 2020). Only few studies were conducted to identify the genetic loci controlling microbiota composition in chicken line. We can mention (Mignon-Grasteau et al., 2015) who identified a total of 14 QTLs influencing the composition of the microbiota. Genetic determinants have been identified in other species, including humans (Nichols and Davenport, 2021). Thus, it can be argued that in all animal species, the composition of the gut microbiome is partly determined by host genetics. Although the microorganisms affected and the host pathways responsible may vary, there is increasing evidence to support the idea that genetically encoded variation in the host determines the abundance of specific taxa present in the body (Ryu and Davenport, 2022).

In addition to host genetic variations, the gut microbiota is likely a driver of the observed variations in vaccine response levels. The gut microbiota in chickens, as well as in all farm animals or in humans, interacts closely with host immunity (Valdez et al., 2014; Zimmermann and Curtis, 2018; Ciabattini et al., 2019), with an impact on animal health. Vaccine responses, being part of the immune response, might be influenced by the gut microbiota composition, but until now few studies have demonstrated it. In poultry, several studies have demonstrated that altering the microbiota can impact vaccine responses. Administration of various antimicrobials at low concentrations to broilers and layers has been shown to affect humoral and cell-mediated immunity in response to various vaccines or viral challenges (Lee and Lillehoj, 2011). In addition, it has been shown that feeding probiotics to broilers and layers through the diet can improve vaccine responses (Molnár et al., 2011; Beirão et al., 2018).

Vaccine response efficacy is likely the result of complex interactions between host genetics, other immunocompetence parameters and gut microbiota composition. In this study, we investigated the effect of the genetic line on microbiota composition, vaccination responses and other immune traits of laying hens, as a first step toward investigating how microbiota and immunity could interact in these lines under the effect of host genetic variations. We used 4 laying hen lines: 2 experimental lines, ND3 and CTR, and 2 commercial lines from the Novogen breeding company, one Rhode Island line (**RIR**) and one White Leghorn line (**LEG**). We were interested in the ND3/CTR lines as a model of genetic selection toward a higher response to NDV vaccination. We hypothesized if this selection impacted other immune parameters and cecal microbiota composition, and how these traits are related.

We were interested in the RIR and LEG commercial lines because they represent the two types of commercial egg production worldwide. The RIR line represents “brown” egg layers, which account for over 50% of the world market, but with major regional disparities, such as the fact that they represent 80% of the European market and more specifically 90% of the French market. The LEG line represents white egg layers, accounting for over 40% of the world market, particularly in the USA, the Middle East and South-East Asia. In addition, the two lines have different egg-laying characteristics, behavior and robustness. The LEG line produces more eggs overall, but they are smaller than those of the RIR line, which is also considered to be slightly more robust than the LEG line (Novogen, 2023; Hy-Line, 2023; Lohmann., 2023).

The main objectives of this study were to: 1) assess the effect of the host genetics on multiple immune parameters including the vaccine response, by comparing genetic lines; 2) study the line effect on the microbiota composition and its potential functions; and 3) discuss the putative links between microbiota and immune system from our results.

## MATERIALS AND METHODS

### Animals and Experimental Design

Four genetic lines of chicken were used for this study. We used 2 experimental lines derived from the same White Leghorn line: ND3, which has been selected for more than 25 yr for a high antibody response to the Newcastle Disease Virus (**NDV**) vaccine, and a non-selected line named CTR (Pinard-van der Laan, 2002). We also used 2 commercial lines: one White Leghorn line (**LEG**) and one Rhode Island Red Line (**RIR**), from the breeder Novogen. A total of 96 hens were used: 24 hens from each of the 4 lines tested.

All animals hatched together at the PEAT unit (INRAE Val-de-Loire Center, Nouzilly, France). The ND3 and CTR eggs were obtained from the PEAT experimental unit where these lines are bred, and the RIR and LEG eggs were supplied by the NOVOGEN hatchery (Hatchery NOVOGEN, Z.I. La Gare d'Uzel 22460 Saint-Herve, France). In order to minimize the possible bias of the possible microbial contamination on the eggshell derived from different facilities, eggs were cleaned at the PEAT hatchery. More precisely, decontamination was achieved by fumigation with a combination of permanganate and formaldehyde. A starter diet was offered to the birds from 1 to 15 d of age and a grower diet was given from 16 to 42 d of age. Diet and water were supplied ad libitum (for feed composition see Additional file 1).

The animals were raised in 4 cages of 23 to 24 hens each without any other animals in the building, with cages being on the intermediate height, contiguous and with no bedding. To facilitate capture and limit stress, the animals were reared in compartments 1 m wide width X 0.5 m length X 0.4 m height. Each animal had a tag at the base of the wing with a unique identifier. The 4 different lines were randomly mixed in every cage at d 1, with a second randomization at d 22 (**D22**) (while maintaining the same distribution of lines per cage) in order to minimize the cage effect as much as possible. The animals were weighted before their vaccination (D22) and before their sacrifice (D42). Blood samples were taken from the venous occipital sinus and transferred in EDTA-coated tubes at D36, D40, and D42 to measure the vaccination response and immune parameters. To extract the plasma, we centrifugated each sample during 10 min at 1,000 g. Animals were killed at d 42 (**D42**) by electronarcosis and cervical dislocation and cecal contents were collected postmortem on each animal. The two caeca were cut longitudinally with scalpels, which were disinfected with alcohol and rinsed in sterilized water between two samples in order to prevent cross-contamination. Cecal contents were removed using unique-usage spatula, in a gentle way in order to prevent the sampling of the mucosa. Contents from both cecal bags of each bird were put in a Petri dish and quickly mixed with the spatula; from this mix, several aliquots of about 200 mg were put into cryotube before flash-congelation at -80°C in liquid nitrogen and stored at −80°C until further analysis.

Animals were raised and sacrificed according to French regulations (APAFIS#29749-2021021018217492 authorization).

### Vaccination and Postvaccination Response

Animals were vaccinated at 3 wk (D22) against NDV by ocular route with the NOBILIS BI MA5-CLONE 30 vaccine. The vaccine response was measured by 2 different techniques: hemagglutination inhibition test (**HAI**), and ELISA against NDV (ID Screen, Innovative Diagnostics).

Antibodies against NDV were quantified in a standard HAI assay_._ Briefly, 25 µL of NDV antigens diluted at 4 HA units (avian paramyxovirus *type*-1, ANSES, Ploufragan) were added to 25 µL of doubling dilutions of plasma and left for 30 min at room temperature before the addition of 25µL of 1% of chicken red blood cells. HAI titers were expressed as log2 of the reciprocal of the highest dilution of plasma causing inhibition of hemagglutination.

Indirect ELISA was also performed for the detection of antibodies against NDV (ID Screen Newcastle Disease Indirect Conventional Vaccines, Innovative diagnostics), following the manufacturer's instructions. Results were expressed as sample to positive ratio (S/P = mean absorbance of test sample − mean absorbance of negative control / mean absorbance of positive control - mean absorbance of negative control).

### Measure of Immune Parameters

The IgA, IgY, and IgM levels were evaluated in plasma samples by indirect ELISAs. In brief, Nunc MaxiSorp 96-well plates were coated overnight at room temperature with 50 µL of goat polyclonal anti-chicken IgA, IgY or IgM antibodies (Cat n° A30-103A, A30-104A, and A30-102A, Bethyl Laboratories, Texas, United States) diluted at 2 µg/mL in carbonate-bicarbonate coating buffer pH 9.6 (Euromedex, France). Washings were performed using a Wellwash Versa instrument (Thermo Fisher Scientific, Massachusetts, United States) by rinsing 3 times with PBS containing 0.05% Tween 20. Blocking was performed by incubating 300 µL/well of PBS containing 1% BSA (Albumine Bovine, Fr V, Euromedex) for at least 24 h at 4°C. The plasma samples were diluted at 1/10,000 for IgA and 1/100,000 for IgY and IgM in PBS BSA 1% and 50µL/well were deposited in the plates after washing. Chicken Reference Serum (Bethyl Laboratories) was used as standard. Seven points of 2 by 2 serial dilutions were performed for standard curves, starting at 200 ng/mL for IgA, 20 ng/mL for IgY and 100ng/mL for IgM. Plates were then incubated for 1 h at room temperature. After washing, 50µL of goat anti-chicken IgA, IgY, or IgM antibodies conjugated to horseradish peroxidase (Cat n° A30-103P, A30-104P, and A30-102P, Bethyl Laboratories) diluted at 10 ng/mL in PBS BSA 1% were transferred to each well. The plate was incubated for 1 h at room temperature. After washing, 50 µL of substrate (Substrate Reagent Pack Cat n° DY999, BioTechne, Minnesota, United States) was added to each well and incubated for 20 min at room temperature. The reaction was stopped with 50 µL of H2SO4 2N (Sigma, Germany), and absorbance was measured at 450nm using a Multiskan FC microplate photometer (Thermo Fisher Scientific). All assays were performed in duplicate. If the differences in optical densities obtained were greater than 20%, the sample was re-assayed. Quality controls at the plate level were also performed, and the samples were reassayed if negative controls on the plates were higher than 0.15 absorbance unit.

Blood cell counting and identification was performed by flow cytometry using a method adapted from (Seliger et al., 2012) and (Bílková et al., 2017). Within 1 h after collection on EDTA-coated tubes, 50µL of blood was mixed with 10 µL of TransFix reagent (Cytomark, United Kingdom) and kept at room temperature for 1 to 3 d before flow cytometry analysis. For analysis of absolute erythrocyte and CD45+ cells count, fixed blood cells were diluted 1/50 in staining buffer (PBS containing 5% of FBS and EDTA 2mM) and 25µL of this solution was further incubated at 4°C during 20 min with 75µL of anti-CD45-AF488 (clone LT40, diluted in staining buffer at a 2.5 µg/mL final concentration, Southern Biotech, Alabama, United States). Antibody and blood cells mix was then diluted again at a 1 to 20,000 final dilution. Total blood cell count and % of CD45+ cells were determined on an easyCyte 6HT-2L Guava flow cytometer (Millipore, France) that is able to measure the exact volume of liquid used during analysis, allowing automatic calculation of absolute cell count without the use of quantification beads. Approximately 30,000 single events and 700 CD45+ cells were recorded for each sample. Absolute cell count was recorded as number of cells per μL of blood sample (re-calculated based on dilutions).

For identification of the different cell subtypes among CD45+ cells, 8 µL of fixed blood cells were stained 100µL of staining buffer containing a combination of antibodies: anti-CD45-SPRD (clone LT40, 0.2 µg/mL), anti-CD4-PE-Cy7 (clone CT4, 0.02 µg/mL), anti-CD8α-AF700 (clone CT8, 0.5 µg/mL), anti-TCRγδ-AF488 (clone TCR1, 1 µg/mL), anti-Bu1-AF647 (clone AV20, 0.5 µg/mL), and Kul01-PE (0.1 µg/mL) (all from Southern Biotech), and anti-integrin αV/β3-AF405 (clone 23C6, 5 µg/mL, Santa Cruz Biotechnologies, Texas, United States). After a 20 min incubation at 4°C, cells were centrifuged (300 *g*, 5min, 4°C), supernatant was discarded, cells were suspended in 200 µL of staining buffer, centrifuged (300 *g*, 5min, 4°C), and supernatant was discarded. Cells were finally fixed in BD CellFIX solution (BD Biosciences, New Jersey, United States) before analysis on a BD LSR Fortessa cytometer (BD Biosciences). The data analysis was performed using FlowJo V10 software. All antibodies used in this study were titrated for optimal signal/noise ratios and single-stained samples were used to calculate a fluorescence compensation matrix. Approximately 30,000 CD45+ cell events were recorded for analysis of relative thrombocyte and leukocyte type counts.

Erythrocytes were distinguished as CD45- cells. Among CD45+ cells, based on forward side scatter (**FSC**) and side scatter (**SSC**), heterophils were characterized as large and mostly granular cells. Monocytes were identified as SSC^med^ KUL01^+^ cells and thrombocytes as SSC^med^ KUL01^−^ integrin αV/β3^+^ cells. Among SSC^low/med^ KUL01^−^ integrin αV/β3^−^ cells, B lymphocytes were identified as Bu1^+^ cells. Among B cells blast cells were identified with a higher FSC and SCC. γδT cells were distinguished as TCRγδ^+^ cells and their expression of CD8α was evaluated. Among the remaining T cells (Bu1^−^ TCRγδ^−^ lymphocytes), double negative T cells (CD4^−^ CD8α^−^), T helper cells (CD4^+^ CD8α^−^), cytotoxic T cells (CD4^−^ CD8α^+^) and double positive T cells (CD4^+^ CD8α^+^) were identified. Absolute cell count of each population was calculated based on the absolute cell count and % of CD45+ cells measured. Heterophils/Lymphocytes (H/L) and CD4/CD8α ratios were also calculated.

### DNA Extraction, Amplification and 16 S rRNA Gene Sequencing

DNA from cecal microbiota samples were extracted at the @BRIDGe platform (INRAE, Jouy-en-Josas). Individual cecal DNA was extracted from approx. 200 mg of frozen cecal contents using a standardized protocol slightly adapted from (Godon et al., 1997). Briefly, samples were incubated at 70°C for 1 h with 250 μL of guanidine thiocyanate buffer [4 M guanidine thiocyanate—0.1 M Tris (pH 7.5) and 40 μL of 10% N-lauroyl sarcosine—0.1 M phosphate buffer (pH 8.0)] and 500 μL of 5% N-lauroyl sarcosine. One volume (750 μL) of 0.1-mm-diameter silica beads (Sigma) was added, and tubes were shaken for 10 min at the maximum speed of a Vibrobroyeur MM200 (Retsch, Germany). Tubes were vortexed and centrifuged at 14,000 rpm for 5 min at 4°C. After recovery of the supernatant, 30 μL of Proteinase K (Chemagic STAR DNA BTS kit, Perkin Elmer, Massachusetts, United States) were added and samples were incubated for 10 min at 70°C at 250 rpm in Multi-Therm (Benchmark Scientific, New Jersey, United States), then for 5 min at 95°C for enzyme inactivation. Tubes were centrifuged at 14,000 rpm for 5 min at 4°C and supernatant was transferred in a deepwell. The plate was transferred on the nucleic acid workstation Chemagic STAR (Hamilton, Perkin Elmer) and the extraction protocol was performed with Chemagic STAR DNA BTS kit (Perkin Elmer) according to the manufacturer's instructions. DNA quantities were evaluated using a Qubit analyzer (Thermo Fisher Scientific), and DNA qualities using a Nanodrop analyzer (Thermo Fisher Scientific) by measuring ratios of absorbances at 240, 260, and 280 nm.

Sequencing was performed by an Illumina Miseq 2*250 bp sequencer by the INRAE @BRIDGe platform, using amplicons of the V3-V4 region of the 16S rRNA gene obtained with the primers Forward PCR1F_343: ACGGRAGGCAGCAG and Reverse PCR1_R784: TACCAGGGTATCTAATCCT. We conducted the first PCR reaction with 1 μL of genomic DNA and 0.5 μL of each primer (10 μM), 0.5 μL of dNTP mix (10 mM), 2.5 μL of 10X MTPtaq buffer mix (10 mM), 0.25 μL of MTP Taq DNA Polymerase (SIGMA-ALDRICH) and H2O qsp 25 μL. PCR conditions were: initial denaturation at 94 °C for 10 min, followed by 30 cycles of 94 °C for 1 min, annealing at 68 °C for 1 min, extension at 72 °C for 1 min and final elongation step at 72 °C for 10 min. Amplicons were then purified using a magnetic beads CleanPCR (Clean NA, GC biotech B.V.,The Netherlands). The concentration of the purified amplicons was controlled using a Nanodrop spectrophotometer (Thermo Scientific) and a subset of amplicons size was controlled on a Fragment Analyzer (AATI, California, United States) with the reagent kit ADNdb 910 (35-1,500 bp). In the second PCR, samples were multiplexed and another pair of primers was used (PCR2_P5F: AATGATACGGCGACCACCGAGATCTACACT and PCR2_P7R: CAAGCAGAAGACGGCATACGAGAT-NNNNNN-GTGACT) with the following PCR steps: an initial denaturation step (94°C for 10 min), 12 cycles of amplification (94°C for 1 min, 65°C for 1 min and 72°C for 1 min) and a final elongation step at 72°C for 110 min. Amplicons were purified and the DNA concentration was controlled as described for the first PCR reaction. One run on an Illumina MiSeq was used to sequence amplicons according to the standard protocol. The raw reads were submitted to the NCBI Sequence Read Archive database (Accession Number: PRJNA1070677).

### Bioinformatics Analyses for Microbiota Description

Raw sequences were analyzed using the R package Dada2 version 1.26 (Callahan et al., 2016, p. 2) on R software (version 4.1.3) (R Core Team, 2022). The FastQC version 0.11.9 (Andrews, 2010) program was used to control sequences quality and the Cutadapt program version 2.5 (Martin, 2011) was used to find and remove adapter sequences from sequencing reads. R1 and R2 reads were merged and filtered with the following parameters: truncate reads at the first instance of a quality score less than or equal to 2 and a maximum number of “expected errors” allowed in a read of 2. The chimeras were removed with the “consensus” algorithm. The recently developed IdTaxa taxonomic classification method was used with the Decipher package version 2.26 (Wright, 2016) and with the Silva SSU r138 trained classifiers (Quast et al., 2013). For all the analyses involving diversity measures, rarefaction was performed using the rarefy_even_depth function in the phyloseq (1.24.2) package. At the end, the Amplicon Sequence Variant (ASV) table was obtained for each individual.

The Phyloseq (1.24.2) (McMurdie and Holmes, 2013) and Vegan (2.5-3) packages (Oksanen et al., 2022) in R were used to perform diversity analyses on the rarefied data. Alpha diversity indices were measured based on the Chao1 and Simpson indexes. Beta diversity indices were calculated with the Bray-Curtis and the Unifrac methods.

The Anova tests were performed to test the significance of differences in alpha diversity indices between lines. Permutational multivariate analyses of variance (Permanova) were used to test for significance of differences in beta diversity indices between lines.

Then, ASV were aggregated by phylogenetic rank with the command tax_glom to identify differentially abundant (**DA**) families and genera. Functional gene families and the KEGG pathways (level 1 and level 2) were predicted using the PICRUST2 software (Douglas et al., 2020). DA KEGG orthologs (KO) and DA KEGG pathways (Release 107.0) were identified using the R package DESeq2 (1.26.0) (Love et al., 2014).

### Statistical Analyses

All statistical analyses were performed using the R software (version 4.1.3) (R Core Team, 2022). Wilcoxon signed rank test was used for all the comparisons of parameters (microbiota or immunity parameters) between the 4 lines. A non-parametric permutational multivariate ANOVA test, implemented in the Vegan package for R (version 2.6.4), was used to test the effects of lines (corrected by a potential Mother and Father effect) on overall community composition on the Bray-Curtis and weighted-UniFrac distances. We searched for associations between individual cecal microbiota at the genus taxonomic rank and the specific immune phenotype of the vaccine response (calculated here by the anti-NDV ELISA test) by calculating Spearman correlations. To do this, we used the normalized abundances from the previous Phyloseq normalization as a starting point. Then, we calculated the Spearman correlation coefficients, and we set a threshold of 0.05 for their adjusted P-values using the Benjamin-Hochberg method. We first performed these correlations on all the animals and then on each of the lines. All the P-values were corrected, using a Benjamini-Hochberg correction. The significance level was set at *P*-value ≤ 0.05.

## RESULTS

We observed almost no mortality during this experiment, with only 1 mortality 2 d before the end of the experiment (CTR line). We observed a male bird erroneously identified as a female in the LEG line, that we removed from further analyses.

### Body Weight and Growth

**Figure 1:**
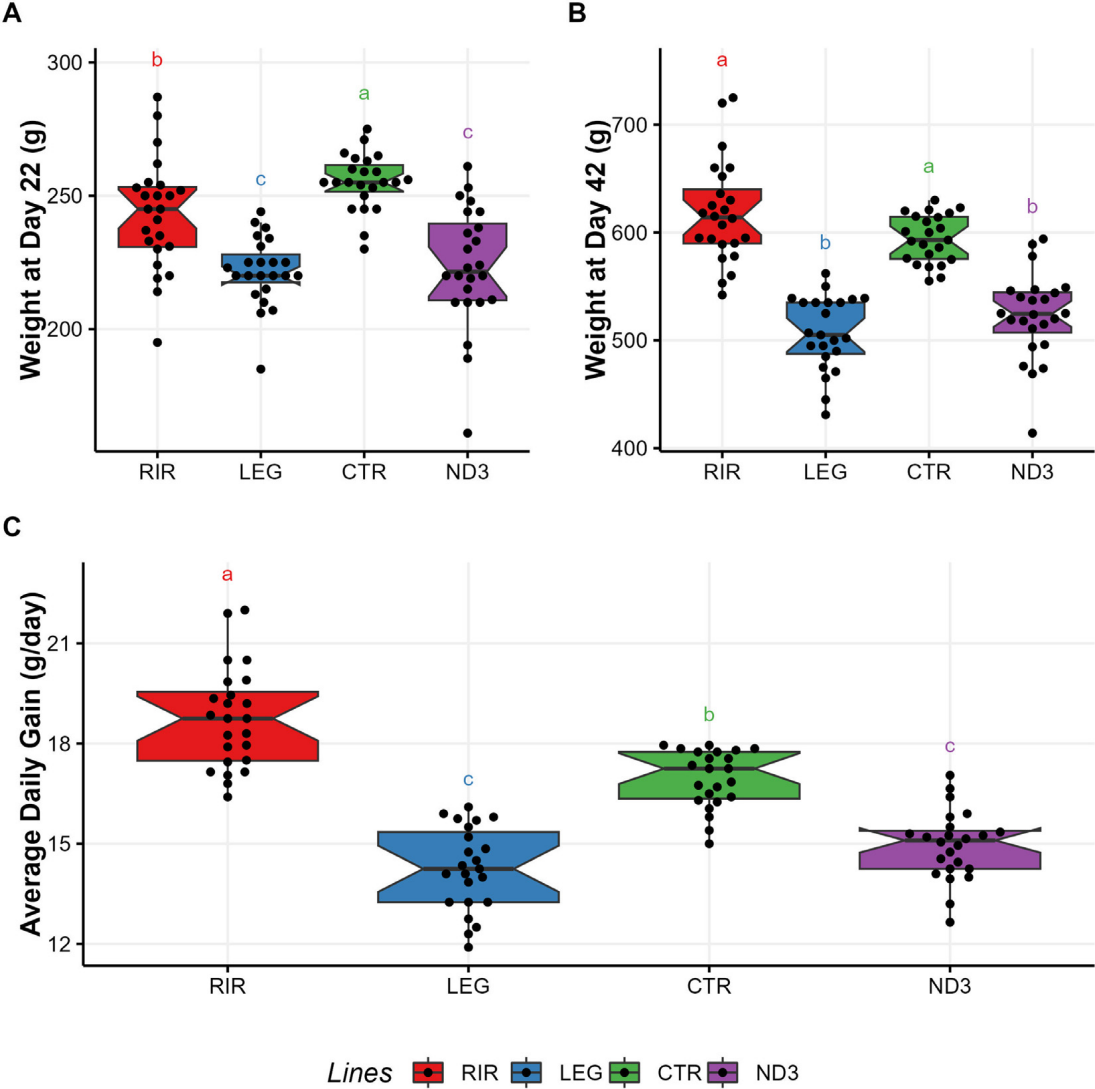
(A) Chicken weight in g at d 22; (B) Chicken weight in g at d 42; (C) Average Daily Gain (g) for the 4 lines. a, b, c: statistical differences considering a *p*-value threshold of 0.05 (Pairwise Wilcoxon test with Benjamini-Hochberg correction).

We weighed the animals at D22 before NDV vaccination and at D42 before the final sacrifice. The results are shown in Figure 1 and supplementary file 1. At D22, the heaviest line was the CTR line (255.22 ± 2.19 g) followed by the RIR line (243 ± 4.28 g); the 2 lines ND3 and LEG showed a similar weight, respectively 223.46 ± 4.61 and 221.78 ± 2.68 g. At D42, the RIR and CTR lines revealed a significantly higher final weight (618.08 ± 9.62 and 594.22 ± 4.68 g, respectively) than the LEG and ND3 lines (506.91 ± 7.19 g and 522.58 ± 8.14 g, respectively).

The RIR line revealed a faster growth than the LEG line (RIR 18.75 ± 0.31 g/d vs. LEG 14.26 ± 0.26 g/d), and the CTR line showed a faster growth rate than the ND3 line (CTR 16.95 ± 0.18 g/d vs. ND3 14.96 ± 0.21 g/d). The selected line ND3 and the commercial line LEG displayed a similar evolution of weight.

### Measure of the Humoral NDV Vaccine Response

**Figure 2:**
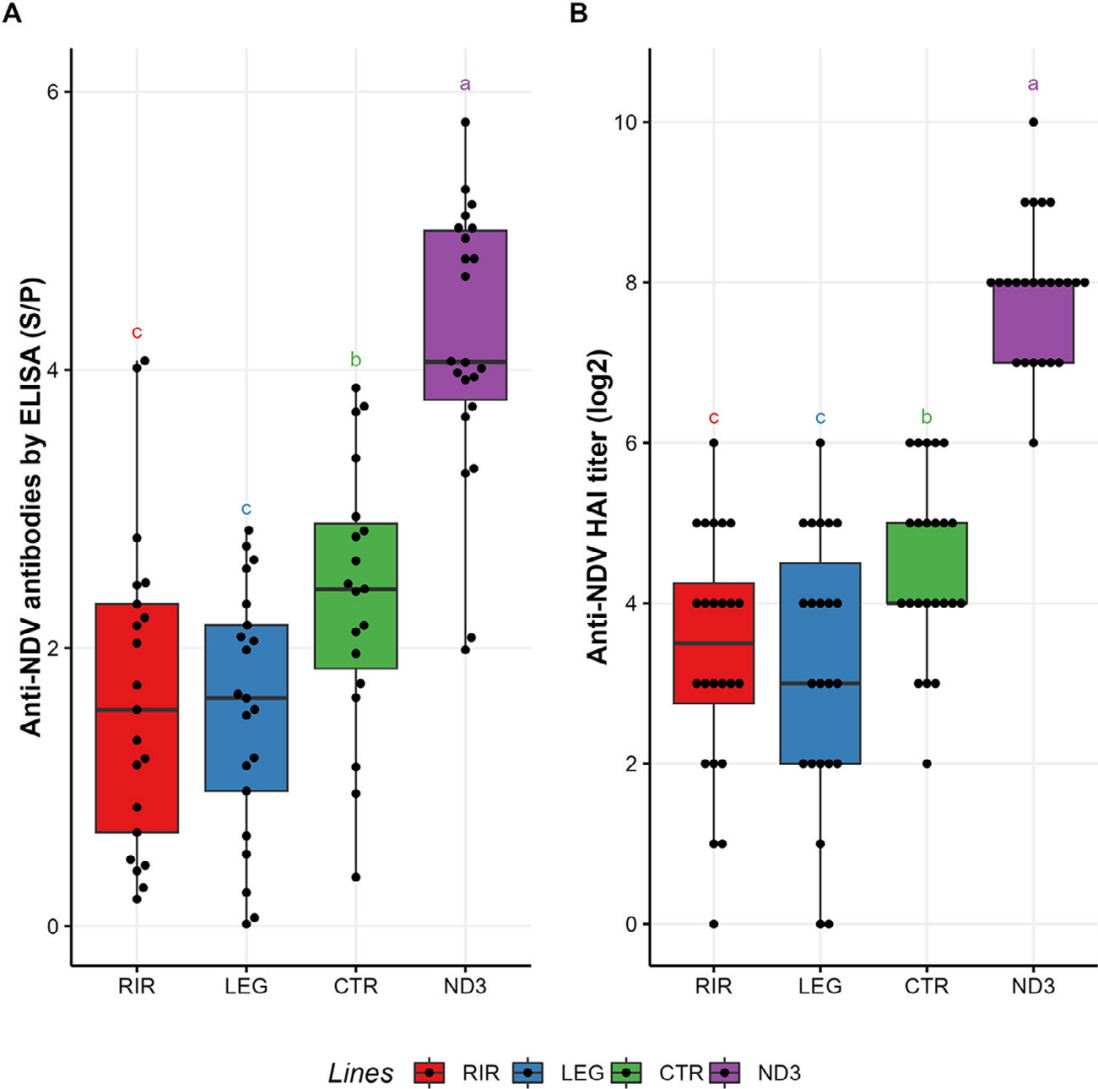
(A) Sample to positive ratio obtained from the ELISA against the Newcastle Disease Virus (**NDV**) antigens, at d 42, for the 4 genetic lines tested; (B) Results of the HAI test against NDV antibody, at d 42, for the 4 genetic lines tested. a, b, c: statistical differences considering a *p*-value threshold of 0.05 (Pairwise Wilcoxon test with Benjamini-Hochberg correction).

Humoral response to vaccination was measured by 2 methods (HAI measuring neutralizing antibodies and ELISA measuring a global antibody response to NDV) (data in Supplementary files 2). We obtained very similar results with the 2 methods used (Spearman correlation of 0.77, *P*-value < 2.2*10^−16^). We observed a very strong line effect (Figure 2), even when correcting by the weight of the animals (*P*-value < 1×10^−14^). ND3 always showed the highest response (HAI titers 7.92 ± 0.18, ELISA S/*P* values 4.21 ± 0.21), the CTR line showed an intermediate level of response (HAI 4.48 ± 0.23, ELISA 2.38 ± 0.22), while the 2 commercial lines displayed lower equivalent levels of response (HAI RIR 3.38 ± 0.31, LEG 3.22 ± 0.34, ELISA RIR 1.66 ± 0.25, LEG 1.55 ± 0.19). This line distribution was similar at earlier time points (measures done at D36 and D40 for HAI and D36 for ELISA) (Supplementary Figure 1).

### Immune Parameters and Blood Composition

**Figure 3:**
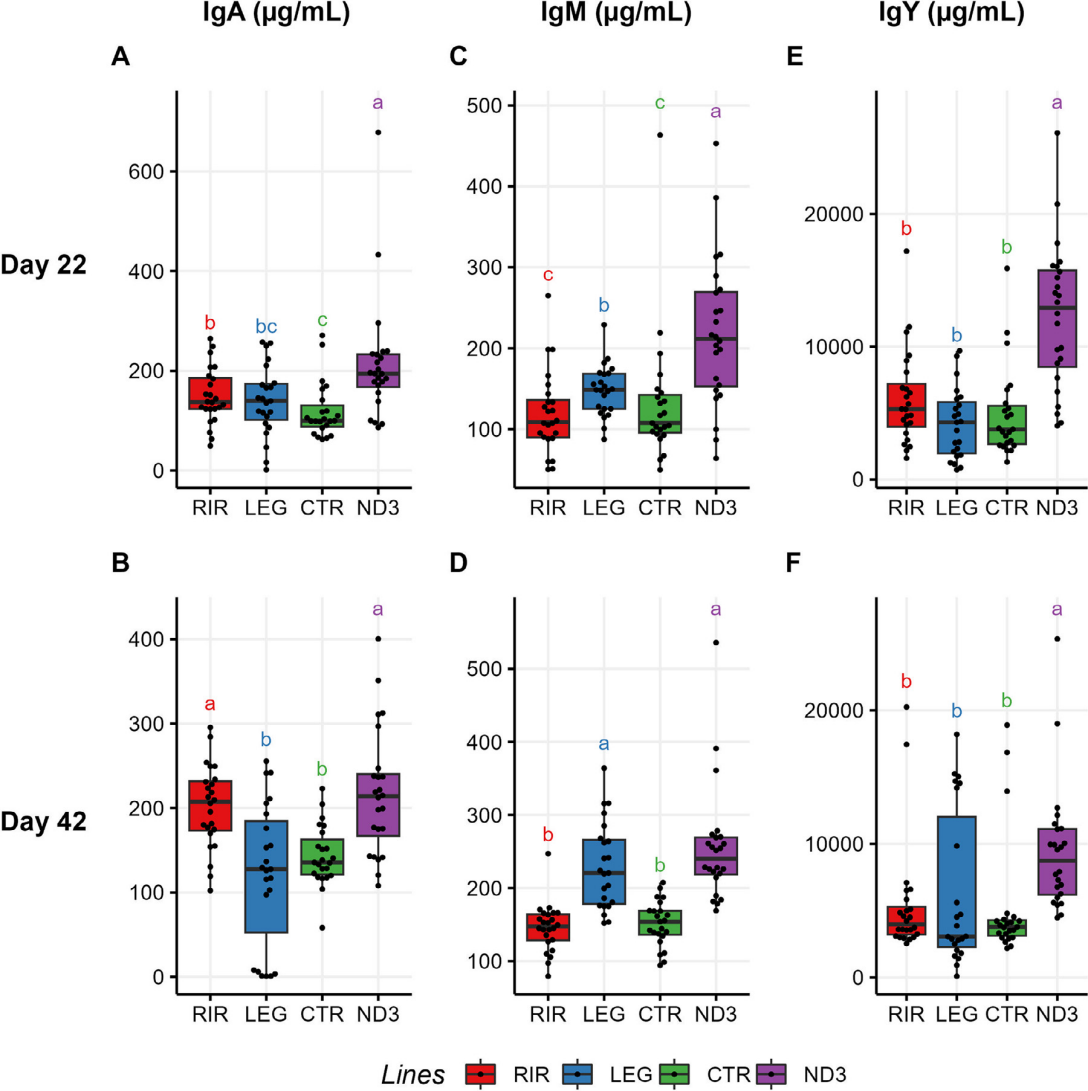
Concentration of seric immunoglobulin A at d 22 (A) and at d 42 (D); Of seric immunoglobulin M at d 22 (B) and (E) at d 42; C) Of seric immunoglobulin Y at d 22 and F) at d 42. a, b, c: statistical differences considering a *p*-value threshold of 0.05 (Pairwise Wilcoxon test with Benjamini-Hochberg correction).

To gain knowledge on the genetic line effect on other immune parameters, we also assessed total IgA, IgM and IgY in plasmas as well as blood cell composition. IgA, IgM and IgY measurements are shown in Figure 3 and Supplementary files 3. We observed that the ND3 line has the highest amount of Ig for the 3 subclasses and at the 2 time points considered (D22 and D42). We also observed increased concentrations of IgA in the RIR line, comparable to the concentration observed in the ND3 line, and a high amount of IgM in the LEG line also comparable to the concentration observed in the ND3 line. Finally, we observed that the CTR line had the lowest amount of Ig (comparable to the commercial lines RIR for IgM and LEG for IgA and RIR and LEG for IgY), and this for all time points and the 3 Ig subclasses.

Results of blood cell composition assessed by flow cytometry analyses at D22 and D42 are shown in Table 1. The differences between the different lines at D42 were first studied using a PCA to represent the different lines according to their blood formulae. This enabled us to see that the 4 lines were very similar at D22, but not at D42, the RIR line stood out quite significantly from the other 3 lines. We therefore wanted to study the differences between the 2 pairs of lines studied at D22 and D42 (RIR/ LEG and ND3/CTR). For the comparison between RIR and LEG, there were only 3 cell types with different abundances (thrombocytes and heterophils were more abundant in RIR and γδ T cells in LEG). The H/L ratio was also higher in the RIR line. On the other hand, at D42, many cell types were significantly more abundant in the RIR line (thrombocytes, leucocytes, lymphocytes, B cells, T cells, CD4^−^ CD8α^−,^ CD4^+^ CD8α^+,^ CD4^−^ CD8α^+^, monocytes and higher percentage of Blast B cells per B cells and γδ T cells CD8α^+^ per γδ T cells). On the contrary, only γδ T cells and CD4^+^ CD8α^−^ T cells were more abundant in the LEG line.

**Table 1:** Blood formula at D22 and D42 for the 4 lines.

	D 22			D 42			D 22			D 42		
	RIR	LEG		RIR	LEG		CTR	ND3		CTR	ND3	
Erythrocytes (10^9^ cells / mL)	28.7 ± 1	31.9 ± 2.3		19.8 ± 0.7	20.6 ± 0.6		30.8 ± 1.7	28 ± 0.9		22.1 ± 0.5	20.6 ± 1.2	
Thrombocytes (10^6^ cells / mL)	**2.4 ± 0.2**	1.7 ± 0.2	**	**2.5 ± 0.3**	1.3 ± 0.1	***	1.8 ± 0.2	1.6 ± 0.2		**1.4 ± 0.1**	1.1 ± 0.1	*
Leucocytes (10^6^ cells / mL)	88.5 ± 5	79.4 ± 5.3		**41.4 ± 1.8**	31.7 ± 1.3	****	72.7 ± 4.2	61.2 ± 3.5		28.6 ± 0.9	27.9 ± 1.1	
Lymphocytes (L) (10^6^ cells / mL)	49.3 ± 5.2	49.2 ± 4		**32.8 ± 1.6**	25.8 ± 1.3	**	41.5 ± 4.6	33.7 ± 3.8		22 ± 0.9	22.8 ± 0.9	
B cells (10^6^ cells / mL)	13.9 ± 1.8	9.3 ± 1		**8.7 ± 0.5**	5.1 ± 0.3	****	7.5 ± 1	6.4 ± 1.2		3.9 ± 0.3	4.5 ± 0.3	
blast B cells (% of B cells)	11.9% ± 0.6	13.1% ± 0.7		**20.2% ± 1.8**	9.6% ± 0.7	****	16% ± 0.8	17.7% ± 0.8		9.4% ± 0.5	9.1% ± 0.5	
T cells (10^6^ cells / mL)	35.4 ± 3.4	40 ± 3.2		**24.1 ± 1.2**	20.7 ± 1.1	*	34 ± 3.7	27.3 ± 2.7		18.1 ± 0.7	18.3 ± 0.7	
γδ T cells (10^6^ cells / mL)	4.6 ± 1.3	**7.3 ± 0.8**	**	3.2 ± 0.2	**5.4 ± 0.3**	****	5.4 ± 0.8	3.6 ± 0.5		**4.2 ± 0.2**	3.3 ± 0.2	**
γδ T cells CD8a+ (% of γδ T cells)	4.2% ± 0.5	3.6% ± 0.5		**4.7% ± 0.4**	2.8% ± 0.4	***	2.7% ± 0.4	2.7% ± 0.4		2.4% ± 0.2	2.4% ± 0.2	
CD4- CD8α- (10^6^ cells / mL)	6.9 ± 0.8	6.6 ± 0.6		**3.9 ± 0.3**	2.5 ± 0.3	***	**4.6 ± 0.6**	3 ± 0.5	*	2.3 ± 0.2	1.9 ± 0.2	
CD4- CD8α+ (10^6^ cells / mL)	17.6 ± 1.9	20.3 ± 1.6		**12 ± 0.6**	9.5 ± 0.5	***	19.6 ± 2.5	16.7 ± 1.7		8.6 ± 0.4	**10.2 ± 0.4**	**
CD4+ CD8α- (10^6^ cells / mL)	2.1 ± 0.3	2.2 ± 0.3		0.3 ± 0.1	**0.5 ± 0.1**	*	1.6 ± 0.3	1.3 ± 0.2		0.7 ± 0.1	0.6 ± 0.1	
CD4+ CD8α+ (10^6^ cells / mL)	25 ± 1.3	20 ± 2.1		**11.5 ± 0.7**	6.3 ± 0.8	****	22 ± 1.6	23.4 ± 2.5		9 ± 0.8	9.3 ± 1.4	
Monocytes (10^6^ cells / mL)	5.4 ± 0.5	5.6 ± 0.8		**2.6 ± 0.2**	1.8 ± 0.1	**	**5.9 ± 0.5**	4.4 ± 0.5	*	**2.2 ± 0.1**	1.6 ± 0.1	**
Heterophils (H) (10^6^ cells / mL)	**33.6 ± 3.2**	24.6 ± 4.4	*	5.9 ± 0.7	4 ± 0.3		24.8 ± 3.6	22.4 ± 2.3		4.3 ± 0.4	3.5 ± 0.3	
CD4+ / CD8α+ Ratio	2.7 ± 0.4	2.8 ± 0.2		3.2 ± 0.2	8.3 ± 2.9		3.6 ± 0.3	**6 ± 1.3**	**	3.6 ± 0.4	**7.4 ± 1.5**	**
H / L Ratio	**1 ± 0.2**	0.8 ± 0.3	*	0.2 ± 0	0.2 ± 0		1.1 ± 0.3	1 ± 0.2		0.2 ± 0	0.2 ± 0	

The “*P* value” columns represent the *P* value of a Wilcoxon test for the 2 comparisons of RIR vs. LEG and CTR vs. ND3 lines at the 2-time points studied (D22 and D42). The erythrocytes are displayed per 10^9^ cells per mL, the other blood cells types per 10^6^ cell per mL.

LEG is a commercial White Leghorn line, RIR a commercial Rhode Island Red line, ND3 a line selected for an higher vaccine response in comparison to a nonselected control line named CTR.

Symbols indicate a significant difference between RIR vs LEG lines or CTR vs ND3 line (* = *P* value ≤ 0.05, ** = *P* value ≤ 0.01, *** = *P* value ≤ 0.001, **** = *P* value ≤ 0.0001) or tendency for a difference (° = *P* value ≤ 0.1).

When comparing the CTR and ND3 lines we found less differences in their blood cell composition. At D22, the CTR line had more CD4^−^ CD8α^−^ T cells and monocytes, while the CD4^+^/CD8α^+^ ratio was higher in the RIR line. Of note, this ratio increased between D22 and D42 in the ND3 line. At D42, we found a higher quantity of thrombocytes, γδ T cells and monocytes in the CTR line, and a higher quantity of CD4- CD8α+ T cells in the ND3 line.

### Gut Microbiota Analysis

#### Sequencing and Global Microbiota Composition

We obtained 4.55 million raw sequences, with an average of 48,892 sequences per sample. At the output of Dada2, the taxonomy table of the 204 individuals contained 2,544,710 sequences, with an average of 34,351 sequences per sample, and 6,122 ASV (Amplicon Sequence Variant) in total. One sample with less sequences was eliminated from the analyses (less than 8,500 sequences). We normalized this dataset to 15,962 sequences per individual. The suppression of this individual, combined with the suppression of the 2 individuals eliminated from the study (1 dead and 1 male) doesn't create an imbalance between the sizes of the 4-line groups.

**Figure 4:**
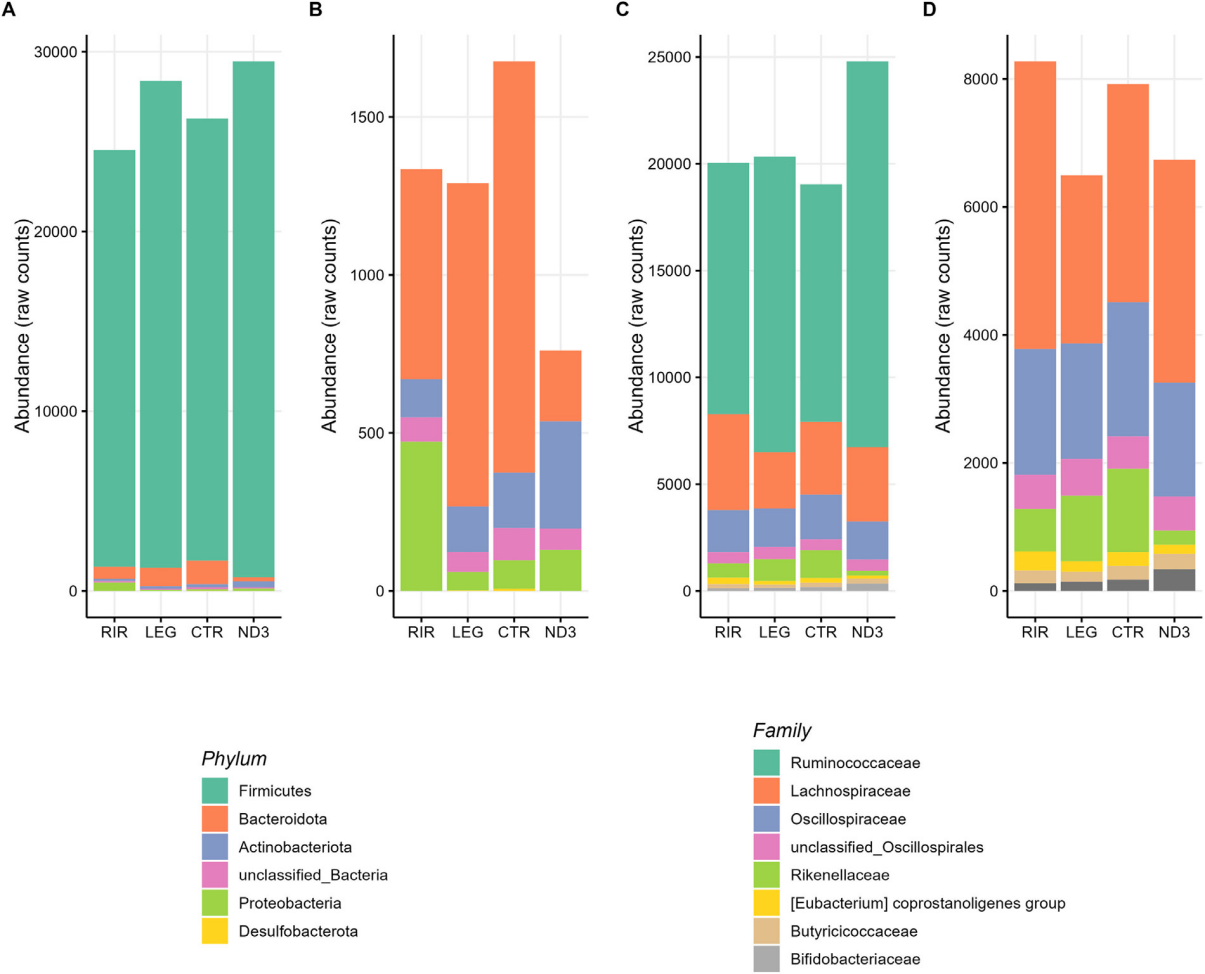
Cecal microbiota composition at phylum and family levels using raw counts. (A) The phylum level, (B) The phylum level without the main abundant phylum Firmicutes; (C) The 8 most abundant families, (D) The 8 most abundant families without the main abundant family Ruminococcaceae.

The cecal microbiota of the 4 lines is largely dominated by the phylum *Firmicutes* (between 93% and 97% of the sequences), followed by *Bacteroidota* (between 0.1% and 2.5%), *Actinobacteriota* (between 0.14% and 0.42%), *Proteobacteria* (between 0.1% and 0.60%), which is in accordance with what is generally observed in the chicken (Figure 4). We also observed some sequences of the phylum *Desulfobacterota* and *unclassified_Bacteria*.

The most represented family was *Ruminococcaceae* (between 41% and 60%), followed by *Lachnospiraceae* (between 8% and 16%), *Oscillospiraceae* (between 5.5% and 8%) and *Rikenellaceae* (between 0.15% and 2.5%) which is also consistent with typical observations in chicken.

#### Alpha and Beta Diversity

**Figure 5:**
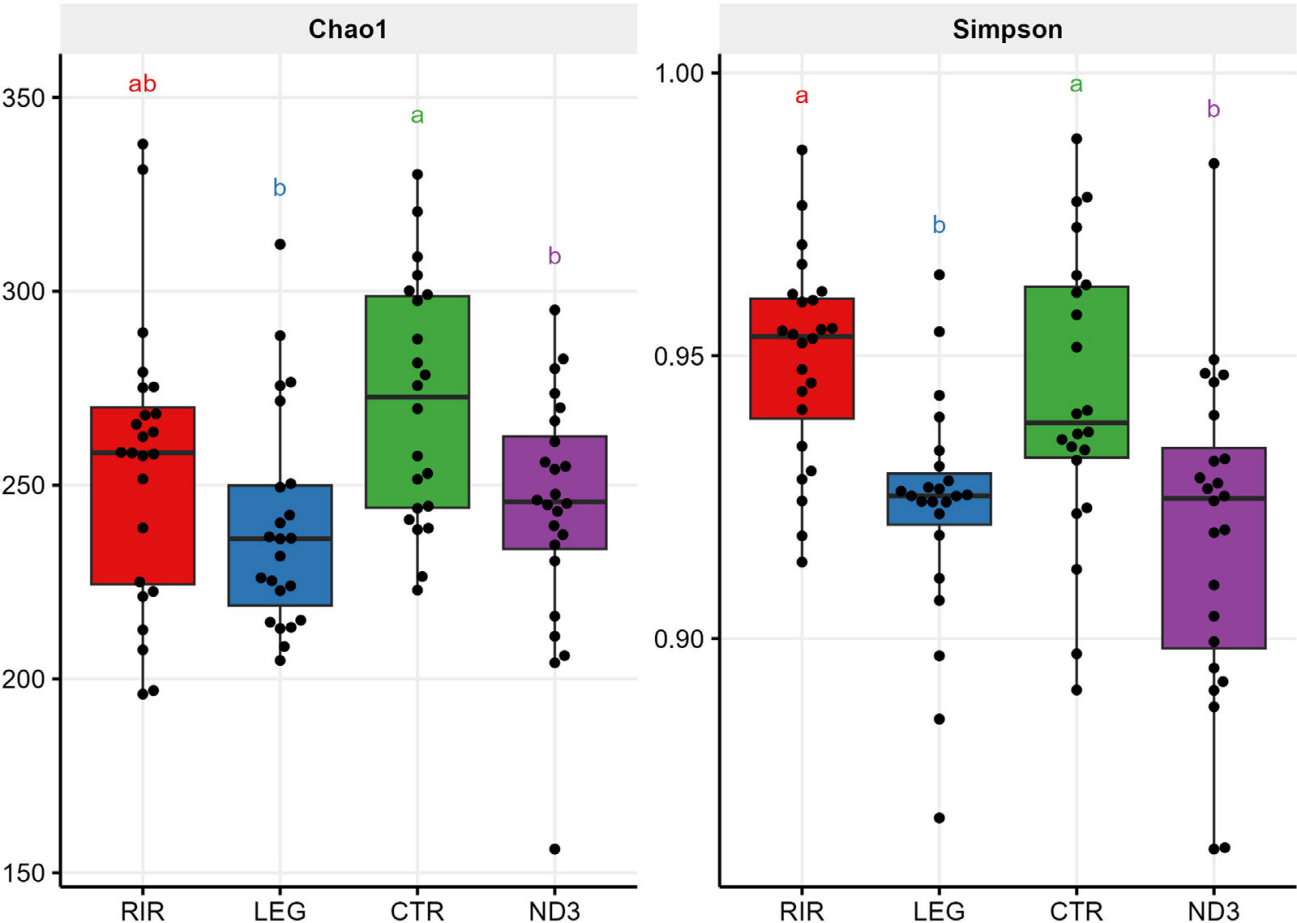
Alpha diversity of the cecal microbiota for each of the tested chicken lines, with Chao1 for the richness and Simpson for the homogeneity of abundance. a, b, c: statistical differences between lines considering a *p*-value threshold of 0.05 (Pairwise Wilcoxon test with Benjamini-Hochberg correction).

We observed a significant difference between lines for the alpha diversity (Figure 5) criteria Chao1: the CTR line (271.45 ± 6.73) was similar to the RIR Line (255.06 ± 7.4), but only CTR was significantly distinct from the other lines. It means that the microbiota of the CTR and RIR lines has a higher number of bacterial species compared to the LEG and ND3 lines (resp. 239.78 ± 5.86 and 244.01 ± 6.18). Comparisons of the Simpson indices also showed significant differences between lines. They were higher in the RIR and CTR lines (resp. 0.95 ± 0.004 and 0.94 ± 0.005) compared to the ND3 and LEG lines (Simpson resp. 0.92 ± 0.006 and 0.92 ± 0.004), thus indicating that the CTR and RIR lines have a more homogeneous microbiota abundance per ASV than the RIR and CTR lines.

**Figure 6:**
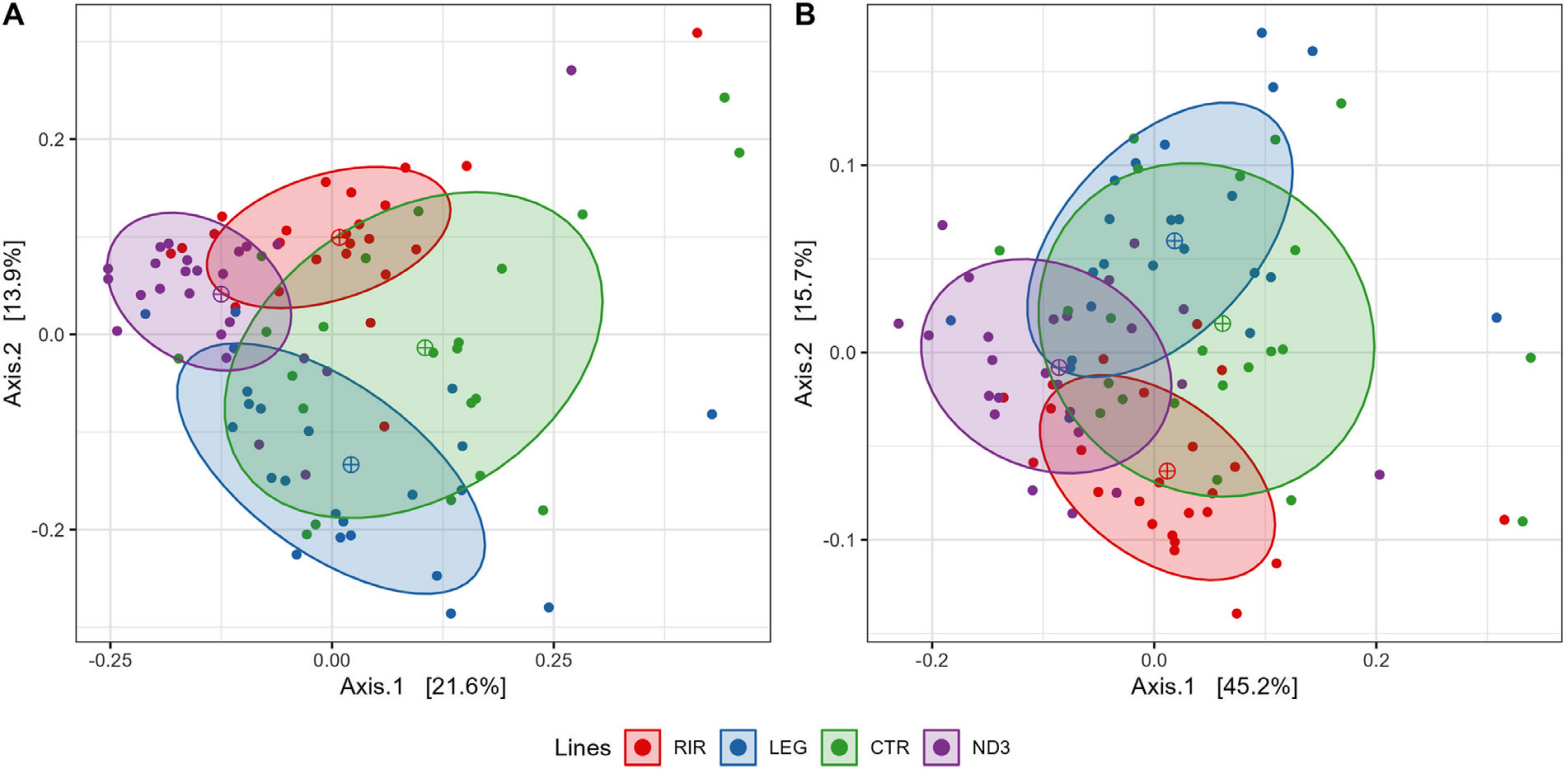
NMDS (nonmetric multidimensional scaling) representation of the cecal microbiota of the (A) Bray-Curtis distance ordination and the (B) Weighted Unifrac distance ordination.

Analysis of beta diversity using Bray–Curtis dissimilarity or Weighted Unifrac with NMDS representations (Figure 6) shows that the microbiota composition significantly clustered by line. The RIR and the LEG lines are the most distant. The Permanova analysis confirmed this line effect (*P* < 0.001) for the 2 methods (Bray-Curtis and Weighted Unifrac). Permanova analyses for all the pairwise line comparisons for the 2 methods showed that all lines were very different from each other, with a *P*-value < 0.001 for all comparisons; except for the LEG / CTR with a non-significant difference of the Weighted Unifrac (*P*-value = 0.071). This showed that the 4 lines are all significantly different from each other. And that the 2 lines with the least distant microbiota compositions were LEG and CTR.

#### Differential Abundances between Lines

We studied the differential abundances in ASV between the 4 lines (6 pairwise comparisons) using the Deseq2 package, at all taxonomic ranks from phylum to genus (Phylum Table 2, other ranks in Supplementary Table). At the genus taxonomic level, we performed an additional test with the condition that the genus must be present in at least one animal from each of the 4 lines (Table 3).

**Table 2:** Log2FC of the pairwise line differential analyses of the cecal microbiota abundance at the phylum rank (non-significant comparisons (*P* value > 0.05) are not shown).

	Log2FC					
Phylum	RIR - LEG	RIR - CTR	RIR - ND3	LEG - ND3	CTR - ND3	LEG - CTR
*Actinobacteriota*			-1,38 *		-1,4 *	
*Bacteroidota*	-1,33 *	-1,18 *	1,3 *	2,63 ***	2,48 ***	
*Desulfobacterota*						
*Firmicutes*	-0,54 *		-0,65 **		-0,75 ***	0,64 *
*Proteobacteria*	2,65 ***	2,33 ***	1,61 **			
*unclassified_Bacteria*	-0,38 *	-0,41 *				

The columns 1 to 3 describe the 3 RIR comparisons, the columns 3 to 5 describe the 3 ND3 comparisons, the last column describes the differences between the lines LEG and CTR.

LEG is a commercial White Leghorn line, RIR a commercial Rhode Island Red line, ND3 a line selected for an higher vaccine response in comparison to a non-selected control line named CTR.

A positive Log2FC represents a higher abundance in the first line of the comparisons. For example, a Log2FC of 2,65 of Proteobacteria in the comparison RIR vs. LEG lines indicates that the RIR line had more Proteobacteria than the LEG line.

Symbols indicate a significant difference (* = *P* value ≤ 0.05, ** = *P* value ≤ 0.01, *** = *P* value ≤ 0.001).

**Table 3:** Log2FC of the pairwise line differential analyses of the cecal microbiota abundance at the genus rank (nonsignificant comparisons (*P* value > 0.05) are not shown).

			Log2FC					
Phylum	Family	Genus	RIR - LEG	RIR - CTR	RIR - ND3	LEG - ND3	CTR - ND3	LEG - CTR
*Bacteroidota*	*Rikenellaceae*	*Alistipes*				2,52 ***	2,65 ***	
*Firmicutes*	*Acholeplasmataceae*	*Anaeroplasma*			2,58 *	4,62 ***	3,61 ***	
	*Erysipelatoclostridiaceae*	*Erysipelatoclostridium*	1,81 ***	1,54 ***	1,59 ***			
	*Erysipelotrichaceae*	*Faecalitalea*		-5,25 *			1,7 ***	
		*Merdibacter*	1,69 *					
	*Enterococcaceae*	*Enterococcus*	4,44 *	4,9 *	5,13 **		0,23 ***	
	*Lactobacillaceae*	*Lactobacillus*	-1,39 **					
	*Streptococcaceae*	*Streptococcus*	4,11 ***	2,95 *	5,24 ***			
	*Christensenellaceae*	*Christensenellaceae R-7 group*			1,39 *	1,75 **	1,71 **	
	*Defluviitaleaceae*	*Defluviitaleaceae UCG-011*	0,74 *					-0,97 *
	*Lachnospiraceae*	*[Ruminococcus] torques group*	1,23 ***		0,77 **			
		*CHKCI001*				1,57 *	1,75 *	
		*Eisenbergiella*	-0,49 *			0,71 ***		
		*GCA-900066575*	-1,07 ***	-0,81 **	-1,01 ***			
		*Sellimonas*	1,92 ***			-1,27 *		
	*Hungateiclostridiaceae*	*Ruminiclostridium*			3,22 **	2,54 *		
	*Oscillospiraceae*	*Flavonifractor*						-0,84 *
		*Oscillibacter*				-0,84 **		
		*unclassified_Oscillospiraceae*	-0,41 *	-0,35		0,48 **	0,42 *	
	*Ruminococcaceae*	*Candidatus Soleaferrea*	-1,97 **	-1,96 **		1,86 **	1,85 **	
		*DTU089*	-1,79 ***	-1,4 ***	-1,27 **			
		*Faecalibacterium*	-0,75 *		-0,9 **			
		*Negativibacillus*	0,81 *					
		*Paludicola*					-1,61 *	
		*Subdoligranulum*	1,58 **			-1,28 *		
	*Anaerovoracaceae*	*Family XIII UCG-001*	1,64 **					
*Proteobacteria*	*Enterobacteriaceae*	*unclassified_Enterobacteriaceae*	2,12 ***	1,75 **	1,55 **			

The columns 1 to 3 describe the 3 RIR comparisons, the columns 3 to 5 describe the 3 ND3 comparisons, the last column describes the differences between the lines LEG and CTR.

LEG is a commercial White Leghorn line, RIR a commercial Rhode Island Red line, ND3 a line selected for an higher vaccine response in comparison to a non-selected control line named CTR.

A positive Log2FC represents a higher abundance in the first line of the comparison.

Symbols indicate a significant difference (* = *P* value ≤ 0.05, ** = *P* value ≤ 0.01, *** = *P* value ≤ 0.001).

At the ASV level, only 82 of the 6,122 ASVs were differentially abundant in at least one of the pairwise comparisons. This is probably due to the nature of ASVs, which require a unique sequence: every bacterial species is composed of different ASVs, each ASV being present in a few individuals. At the family and genus ranks, we logically observed proportionally more differences in abundances.

At the phylum level (Table 2), which is the highest taxonomic rank, some phyla were differentially abundant between lines. In particular, the RIR line had more *Proteobacteria* than the 3 other lines, while the ND3 line had less *Bacteroidota* than the other lines. The ND3 lines had also significantly more *Actinobacteria* than the CTR and RIR lines, but not statistically compared to the LEG line (*P* value = 0.095).

At the genus level (Table 3), which is the lowest taxonomic rank studied, some genera were differentially abundant between lines, with a total of 28 genus DA in at least one line comparison. In particular, the RIR line had more *Erysipelatoclostridium***,** *Enterococcus***,** *Streptococcus* and *unclassified_Enterobacteriaceae***;** and less *DTU089* and *GCA-900066575* than the 3 other lines, while the ND3 line had less *Anaeroplasma, Christensenellaceae R-7 group* than the other lines. Two other genera were significantly less abundant in ND3 vs. 2 lines and trending for the third: *Alistipes* significant versus LEG and CTR and not statistically against RIR (*P* value = 0.070), and *Ruminoclostridium* versus RIR and LEG and not statistically against CTR (*P* value = 0.053).

Differences at other taxonomic ranks are shown in supplementary table. The DA class and order rank were quite similar to the phylum rank, and the genus rank was quite similar to the family rank, but a little more informative.

#### Pathway Inference (Picrust2)

By using the PICRUST2 software, 5488 KEGG orthologs (KO) were predicted based on the ASV table. We used the 133 pathways level 1 of the KEGG database and 25 pathways level 2.

**Figure 7:**
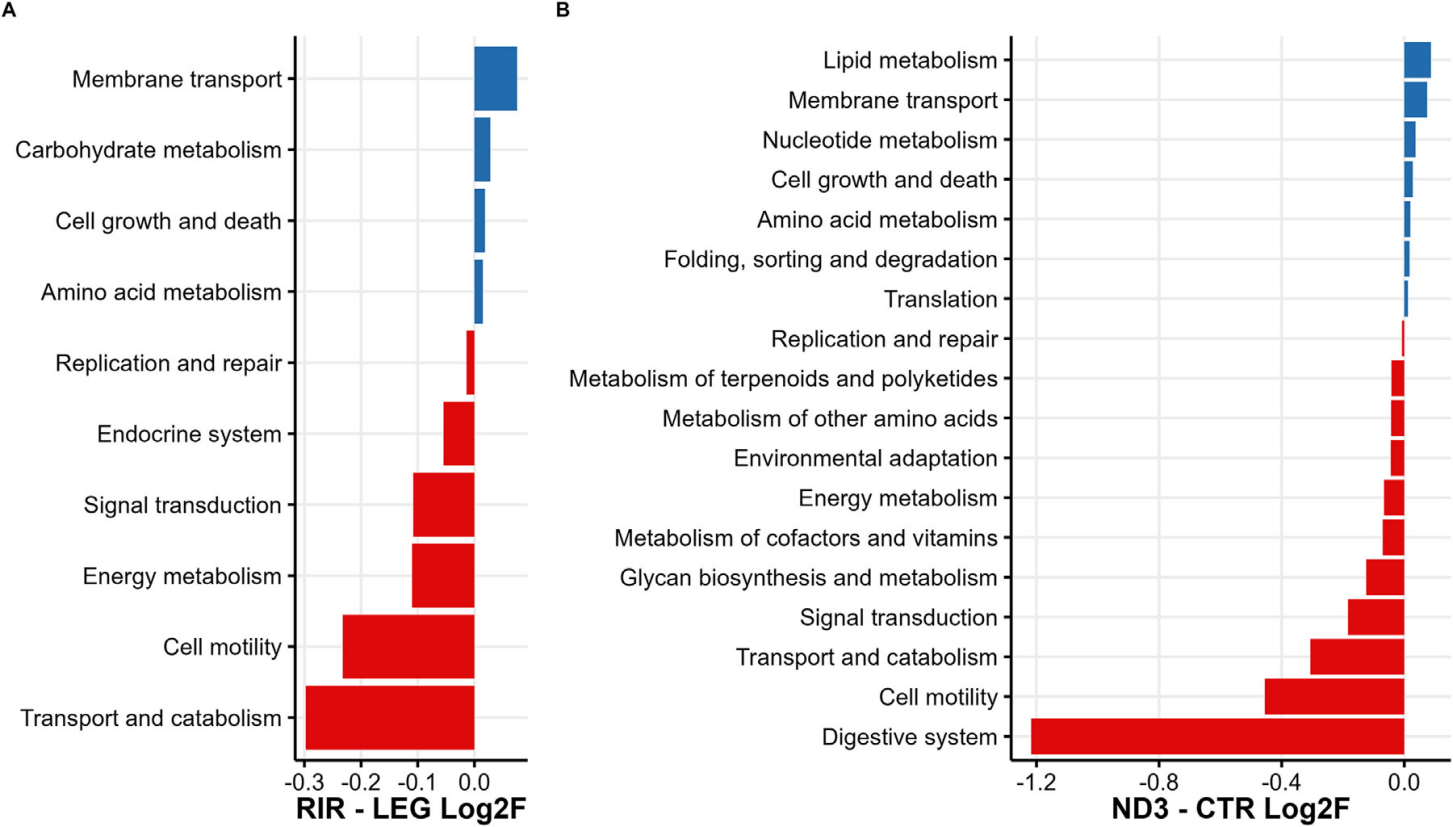
Differentially abundant level-2-pathways from the KEGG database, (A) between the RIR and the LEG lines and (B) between the ND3 and CTR lines. From the pathway enrichment performed by Picrust2.

The DESeq2 package identified 3,137 differentially abundant (DA) KO, 47 DA level-1 pathways and 10 DA level-2 pathways between lines RIR and LEG; and 1,762 DA KO and 75 DA level-1 pathways and 18 DA level-2 pathways between lines ND3 and CTR [all the pathways level 1 and level 2 are shown in the Supplementary Table 2]. Figure 7 shows the DA level 2 pathways identified for RIR / LEG and ND3 / CTR.

The comparison between RIR and LEG lines showed that in the RIR line the pathways “Membrane Transport” and “Carbohydrate metabolism” are up-regulated while the pathways “Transport and catabolism” and “Cell motility” were down-regulated. Pathways with the highest differences in abundance in the ND3/CTR comparison were “Lipid metabolism” and “Membrane transport”, more abundant in ND3; and “Digestive System” and “Cell motility”, more present in CTR.

### Correlations Between Microbiota Components and Vaccine Response Parameters

**Figure 8:**
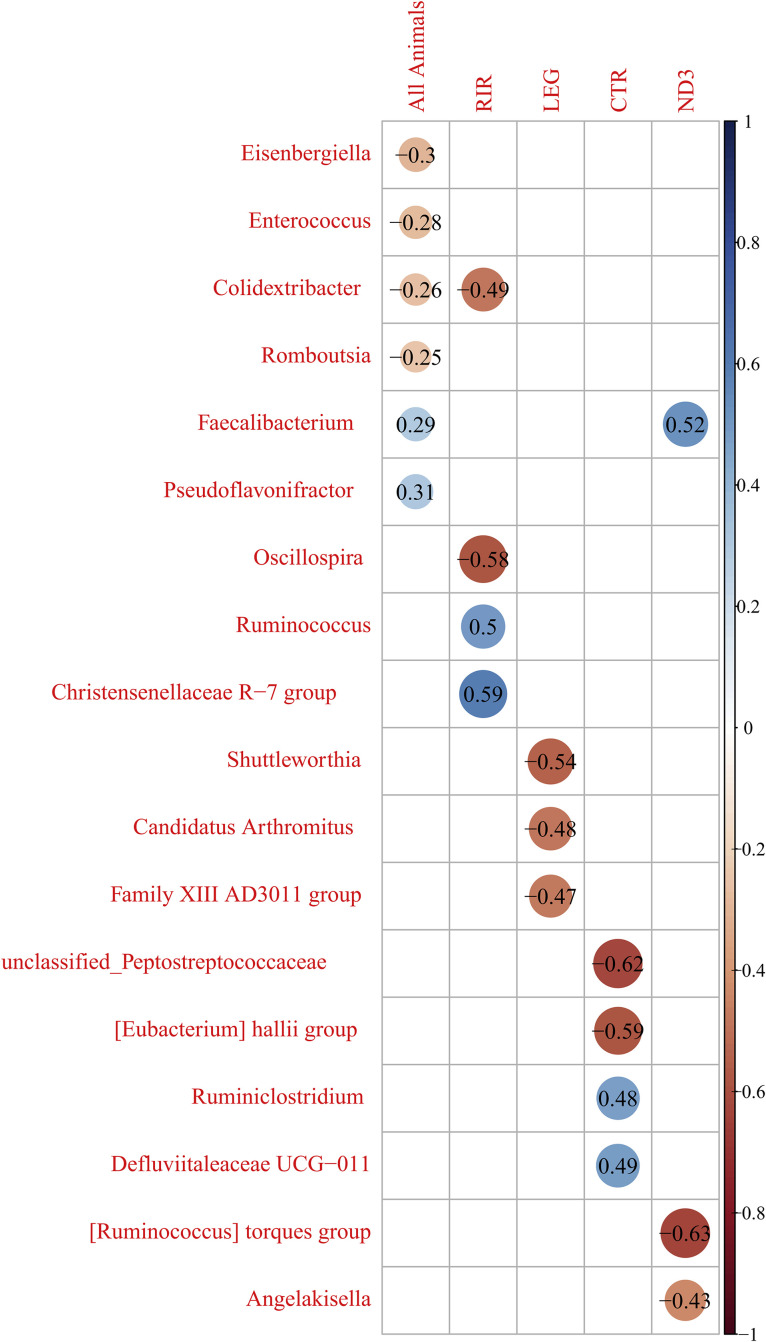
Spearman correlations between the genus and the ELISA NDV-antibody response at D42, for the 4 lines together (all animals), and for each of the 4 lines for the fourth other. Only significant correlations are reported (*P* value < 0.05)*.*

We calculated the correlations between the different bacterial genera observed and the vaccine response levels (here represented by the ELISA at D42) (Figure 8). We used the same list of genera with a minimal presence in at least one animal from each of the 4 lines.

We observed that the abundances of several genera strongly correlated with the vaccine response, in a line-specific way. None of the genera correlated with the vaccine response for the 4 lines considered independently. However, 2 genera correlated significantly with the vaccine response by taking all animals (all lines) into account, but not within a particular line: *Eisenbergiella* (-0.3), *Enterococcus* (-0.28), *Romboutsia* (-0.25) and *Pseudoflavonifractor* (0.31). In other words, all the correlations between one genus and the vaccine response were significant for only one line; some of these correlations were also observed by considering the whole cohort (animals from the 4 lines considered together). The *Colidextribacter* genus significantly correlated with the vaccine response within the RIR line (−0.49), as well as for the whole cohort (−0.26). The *Faecalibacterium* genus correlated significantly and positively with the vaccine response within the ND3 line (0.52) and for the whole cohort (0.29). Three genera correlated significantly with the vaccine response within the RIR line only: *Oscillospira* (−0.58), *Ruminococcus* (0.5) and *Christensenellaceae* (0.59). Three other genera correlated significantly with the vaccine response within the LEG line only: *Shuttleworthia* (−0.54), *Candidatus Arthromitus* (−0.48) and *Family XIII AD3011 group* (−0.47). Four other genera correlated significantly with the vaccine response within the CTR line only: *unclassified_Peptostreptococcaceae* (−0.62), *[Eubacterium] hallii group* (−0.59), *Ruminoclostridium* (0.48) and *Defluviitaleaceae UCG-011* (0.49). Two other genera correlated significantly with the vaccine response within the ND3 line only: *[Ruminococcus] torques group* (−0.63) and *Angelakisella* (−0.43).

## DISCUSSION

We compared 4 different laying hen lines: 3 White Leghorn lines (LEG, ND3, CTR), and one Rhode Island Red line (**RIR**). Two of these lines are commercial lines (RIR, LEG), while the 2 other lines are experimental lines (ND3, CTR). Originating from the same base population, one of the experimental lines has been selected for a high antibody response to NDV vaccine for over 25 yr (line ND3), while the other one (**CTR**) has been maintained without selection (Pinard-van der Laan, 2002). We used this genetic diversity to study the impact of the host genetic background on cecal microbiota composition and immunity, including the vaccine response. The ND3/CTR lines are an interesting and unique model, allowing to investigate the effects on multiple parameters of an efficient selection led on the vaccine response level against NDV. On the other hand, the lines LEG and RIR are more representative of the currently raised lines worldwide, so that their comparison yields result more connected with the real conditions encountered in the poultry industry.

All the animals were raised together, in the same conditions and were fed the same way. Animals from the 4 lines were distributed randomly after their transfer from the hatchery to the rearing building, with a second randomization at D22 in order to minimize the cage effect. In addition, the eggs of the commercial lines were sent to the same hatchery as those of the experimental lines, and from incubation onwards, all the eggs of the 4 lines were processed together. This allowed us to reduce as much as possible the environmental effects that could have distorted our comparisons. We are therefore confident that the differences observed between lines are not due to confounding environmental effects.

### Body Weight and Vaccine Response

The body weight evolution of the ND3 and CTR lines was similar to the evolution mentioned in a previous study we led (Zerjal et al., 2021), in which it was also observed that the weight and the daily weight gain of the CTR line were superior to those of the ND3 line. As mentioned in Zerjal et al. (2021), the reduced growth and body weight of ND3 compared to CTR, could result from previous trade-off accumulated over time, as the ND3 line has been selected for many years with the unique aim of increasing the vaccine response to NDV which in balance could lead to a reduction in body weight. The weight data of the commercial lines were also similar to data acquired independently by the breeder NOVOGEN (Commercial Production Charts). The RIR and the LEG lines are 2 commercially selected lines, with a high egg production, unlike the ND3 and CTR lines, which have not been selected for growth-related parameters nor egg production for almost 30 yr; i.e. since the beginning of the selection experiment.

The vaccine response data of the selected line ND3 (HAI 7.92 ± 0.18) and of the unselected line CTR (HAI 4.48 ± 0.23) was within the known and expected ranges. We previously (Zerjal et al., 2021) obtained HAI titers of 6.6 ± 0.3 for the ND3 line and 4.3 ± 0.3 for the CTR line. It may seem surprising that the commercial lines LEG and RIR show a weaker vaccine response than the non-selected experimental line (CTR). The original commercial line from which ND3 and CTR were derived had maybe a higher vaccine response level than the LEG and RIR lines. Another hypothesis is that the level of vaccine response in commercial lines has declined during the last decades, perhaps as an undesired consequence of selection primarily geared towards egg production. This undesirable phenomenon could be explained by the resource allocation theory (Beilharz et al., 1993), according to which, when an animal is genetically oriented towards high production and efficiency, fewer resources remain for other traits, including immunity or vaccine response (Bayyari et al., 1997; van der Most et al., 2011).

Secondly, concerning the immune data related to the blood cell composition of the animals, we can compare our results to those of our 2 previous studies led on the ND3 and CTR lines (Minozzi et al., 2007; Zerjal et al., 2021). However, the experimental setup of these 2 studies display notable differences with our experiment, so that comparisons should be interpreted cautiously. The first experiment was carried out on crosses of the ND3 line with another line selected for a high cell-mediated immune response, measured using the 24-h delayed-type hypersensitivity response to the mitogen phytohemagglutinin (PHA), that was not used here (Minozzi et al., 2007). The second experiment assessed the blood cell composition at 18 weeks, against 6 weeks for our study (Zerjal et al., 2021). Similarly to (Minozzi et al., 2007), we found at D42 significant differences between ND3 and CTR for the concentration of monocytes and γδ T cells, with a higher concentration in the CTR line. On the other hand, in comparison to (Zerjal et al., 2021), the differences between T cells, B cells, heterophils and CD8 T cells observed at D126 were not observed in our study at D42. This may be due to the fact that these differences appear lately, after D42. Finally, the concentration of CD4+ helper T cells was similar between the 2 lines at D126, whereas we found a significantly higher concentration in the ND3 line at D42. Here again, it is possible that this difference disappears later, leading to the results observed in our previous study.

We can also assume from our data that the RIR line has a blood composition with globally more immune cells, of several different types, as well as more "activated" cells, since the percentage of Blasts B cells per B cells is 20.2%, more than twice that of the LEG line (9.6%) (Nutt et al., 2015).

### Gut Microbiota

The phylum rank is the highest taxonomic rank for describing microbiota composition, while the genus rank is the lowest in the context of our study. We did not infer bacterial species from our sequences because we considered that the degree of confidence provided by 16S sequencing for laying hen gut microbiota was not sufficient to go as far as bacterial species. Considering the ASV rank, we took only limited account of it. Each ASV requires a unique sequence. Consequently, every bacterial species is composed of different ASVs, each ASV being present in a few individuals. Different individuals can therefore carry the same bacterial species but different ASVs. We estimated that the family and genus ranks gave us more relevant information.

We observed differences in global microbiota characteristics between lines. We observed a richer but more unbalanced microbiota in the RIR and CTR (commercial) lines compared to the LEG and ND3 (experimental) lines. Moreover, the 2 indices of beta diversity used (Bray-Curtis and Weighted Unifrac) affirm that the microbiota compositions of the 4 lines are different. We observed significant differences in cecal microbiota composition between lines at all taxonomic ranks, even at the highest taxonomic rank, the phylum. For example, the RIR line has more *Proteobacteria* than the other 3 lines and the ND3 line has more *Actinobacteria* than the other 3 lines. The microbiota compositions of the LEG and CTR lines, although different, are the least distant. We housed animals from different genetic lines in adjacent cages within the same building and using the same diet in our experimental model, with animals from different lines mixed within the cages. Therefore, the differences observed between lines cannot be attributed to contrasted exposures to environmental microbes. We rather hypothesize that differences in host genetic backgrounds led to differences in cecal microbiota composition. This has already been demonstrated for other lines of birds (Borey et al., 2020; Lyte et al., 2021; Chrzastek et al., 2021; Cazals et al., 2022; Sztandarski et al., 2022) but never between the experimental lines ND3 and CTR.

Regarding the pathways inferred from taxonomic compositions of microbiota, the most differentially abundant pathway between the ND3 and CTR lines is the "Digestive System," more present in the CTR line. This observation is consistent with the fact that the CTR line has a higher body weight and growth than the ND3 line. Therefore, it can be assumed that in the conditions of our selection experiment (Pinard-van der Laan, 2002), selection for the vaccine response had as a side effect a decrease, not only in weight and growth, but also of the "digestive" activity of the microbiota. More precisely, the differentially abundant pathway between the ND3 and CTR lines is the “Protein digestion and absorption” pathway. This suggests that the CTR line might display a better protein absorption and digestion due to its distinct microbiota, thus leading to a higher growth (Diaz Carrasco et al., 2019; Su et al., 2021). No causal relationship can be inferred from these results: is the decreased growth observed in ND3 / CTR the consequence of a change in microbiota activity resulting from a shift in the cecal microbiota composition? Are changes in microbiota composition between ND3 and CTR a direct consequence of the selection led on the vaccine response. i.e. are there host genes controlling gut microbiota composition that could have been directly or indirectly selected?

### Link Between Microbiota and Vaccine Response

Since ND3 has been strongly selected for a higher NDV vaccine response, the comparison between ND3 and its control line CTR is the most relevant to study the relation between microbiota composition and vaccine response. Furthermore, the fact that the taxa differentially abundant between RIR and LEG are not the same as for the ND3 and CTR lines is consistent with a specificity of the microbiota related to selection on the vaccine response.

The differentially abundant phylum between the ND3 and CTR lines are *Actinobacteria, Firmicutes* and *Bacteroidota*. In humans, *Actinobacteria* were shown to positively correlate with adaptive immune responses to systemic [Bacillus Calmette–Guérin (**BCG**), tetanus toxoid (**TT**), and hepatitis B virus (**HBV**)] and oral (polio) vaccination in Bangladeshi infants (Huda et al., 2014). Furthermore, it was shown that infants and adults with a greater proportion of the phylum *Firmicutes* in their gut microbiome display increased humoral and cellular responses to oral vaccines (Eloe-Fadrosh et al., 2013; Harris et al., 2017). Conversely, infants with a higher proportion of the phylum *Bacteroidetes* in their gut microbiome exhibit diminished humoral responses to oral vaccines (Harris et al., 2017). Finally, it has been shown that a decrease in the family *Ruminococcaceae* can be associated with an increase in inflammation in humans (Duboc et al., 2012), and even in the chickens (Rychlik, 2020). It therefore appears that the observed increase in *Actinobacteria, Firmicutes* and *Bacteroidota* we observed in animals displaying a higher vaccine response is consistent with previous results obtained in studies led on human populations or chicken.

Regarding the other taxonomic ranks studied (Family and genus), although several DA taxons were identified, none of them had been previously associated with vaccine response or immunity. This does not imply that these taxons have no relation at all with the vaccine response. Only a few studies were led previously on this topic in chickens.

In order to identify possible correlations between taxons and the intensity of the vaccine response regardless of the genetic line, we measured correlations between components of the microbiota (represented by the taxonomic rank of the genus) and the vaccine response to NDV calculated by an indirect ELISA test. Interestingly, the identified correlations are line dependent, since none of the genus studied have significant correlations in the same direction with the 4 lines. Thus, the genera with the best correlations (ranging from −0.5 and −0.63 and 0.49 to 0.59) are "*Colidextribacter*" and *“Christensenellaceae R-7 group”* for the RIR line, *“Shuttleworthia*" for the LEG line, "*[Eubacterium] hallii group*” and “*Defluviitaleaceae UCG-011″* for the CTR line and *“[Ruminococcus] torques group”* and “*Faecalibacterium”* for the ND3 line. Such interactions between microbiota x vaccine response correlations and genetic lines have also been identified in a previous study we led on different genetic lines of hens infected with IBV (Borey et al., 2022). These results show that the variability of microbiota within a line is associated with the level of vaccine response, and that the type of bacteria associated with the vaccine response level varies according to the line. Although we cannot rule out that these correlations are coincidental, it is also worth considering that a causality might exist between the abundance of specific taxons and the intensity of the vaccine response. Two directions of causality have been demonstrated in broiler chicken between microbiota and vaccine response: impact of the microbiota on the vaccine response (Molnár et al., 2011) and impact of the vaccine response on the microbiota (Das et al., 2021). In one case, the composition of the microbiota before vaccination impacts the intensity of the vaccine response, whereas in the other case the vaccine response level impacts the composition of the microbiota after vaccination. Our study does not allow to draw such a conclusion, because we do not know individual microbiota compositions before vaccination, and microbiota composition before vaccination is variable. Considering the specificity of the bacteria associated with the vaccine response according to the line, it is conceivable that different bacteria can achieve the same functions. Functions carried by bacteria might be more important than the bacteria taxonomy.

Since the vaccine response to NDV is statistically similar between the RIR and LEG lines, taxa that are differentially abundant between these lines are not involved in the differences in vaccine response. This is why this comparison is less relevant than the ND3/CTR comparison with regards to the NDV vaccine response.

Although RIR and LEG do not differ for their NDV vaccine response, they displayed a lot of striking differences in their immune parameters, in particular blood cell composition. The observed microbiota composition differences between these lines could be related to these immunological differences. This study provides new DA taxons potentially involved in this relationship.

Studying vaccine response is a complex task due to the diversity of pathogens and vaccines used. In order to gain a more comprehensive understanding of vaccine response, it would be beneficial to investigate multiple vaccines, especially those of different kinds and with various modes of administration, for different diseases. Additionally, since the study focuses on laying hens, it is essential to examine their vaccine response over a prolonged period, including the peak of vaccine response and vaccine persistence.

This study investigates the complex interaction between host genetics, immune responses, and gut microbiota in laying hens, particularly focusing on the response to NDV vaccination. We compared 2 experimental chicken lines (ND3 and CTR) with 2 commercial lines (RIR and LEG), revealing a significant influence of the genetic background on the vaccine response and on the gut microbiota composition. Variations in the gut microbiota across the different chicken lines were observed, suggesting a complex interaction between the host genetics and microbiota. The study also revealed a line-dependent association of microbiota with the vaccine response level, indicating that different bacterial communities may modulate vaccine responses in different genetic backgrounds. In summary, our findings highlight the intricate connections between genetics, immune response, and microbiota in laying hens. These insights can guide future strategies for improving vaccine effectiveness through genetic selection and microbiota management, contributing to more effective disease prevention in poultry production.

## Appendix Supplementary materials

Image, image 1

Image, image 2

Image, application 3

## Footnotes

Supplementary material associated with this article can be found in the online version at doi:10.1016/j.psj.2024.103609.
