# Supplementary figures and images for: Host genetics drives differences in cecal microbiota composition and immune traits of laying hens raised in the same environment
Source: Poult Sci. 2024 Mar 6;103(5):103609. doi: 10.1016/j.psj.2024.103609 (PMC11000118; doi:10.1016/j.psj.2024.103609)

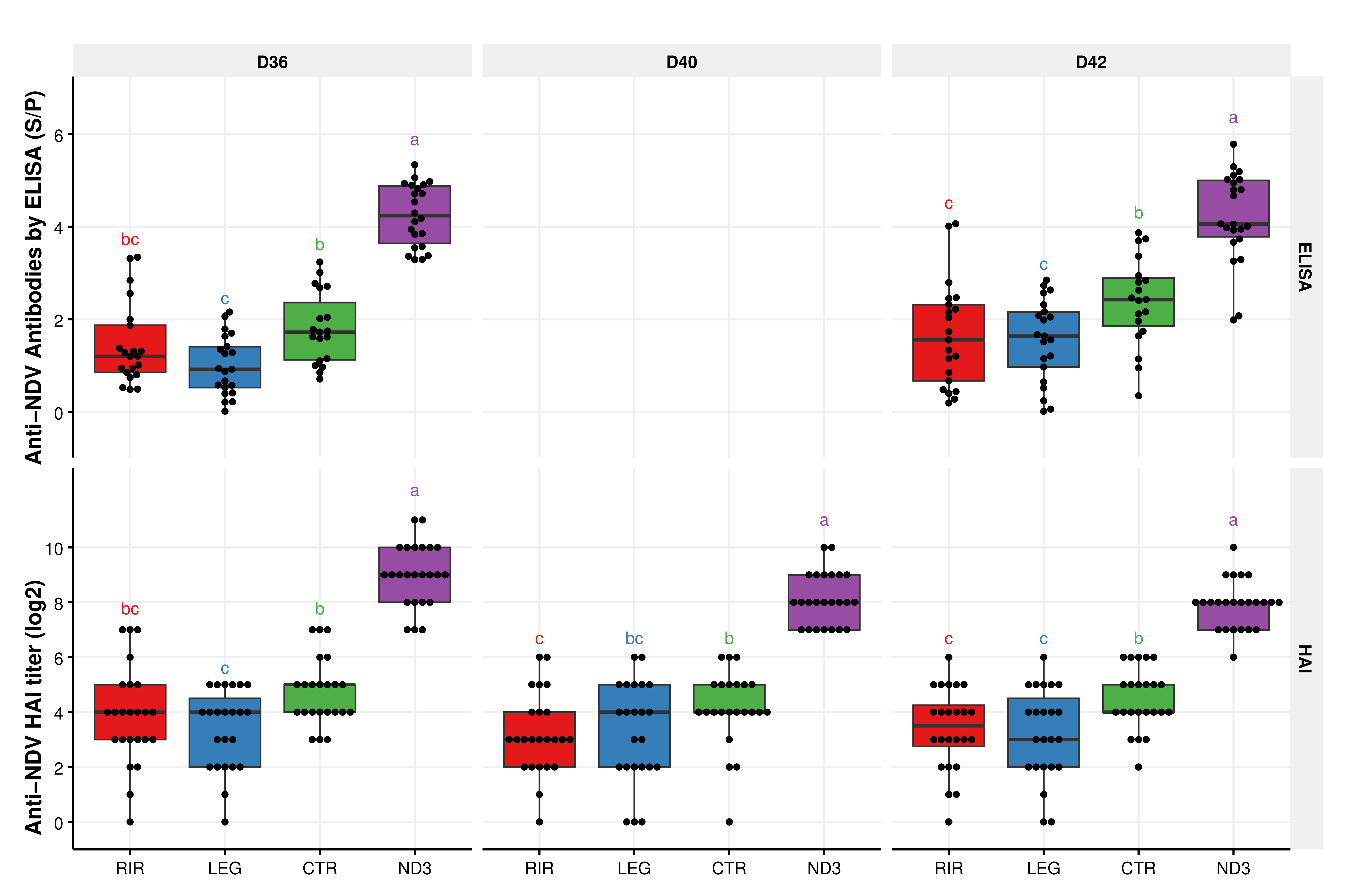

Supplement: Supplementary file 1 [file mmc1.jpg]

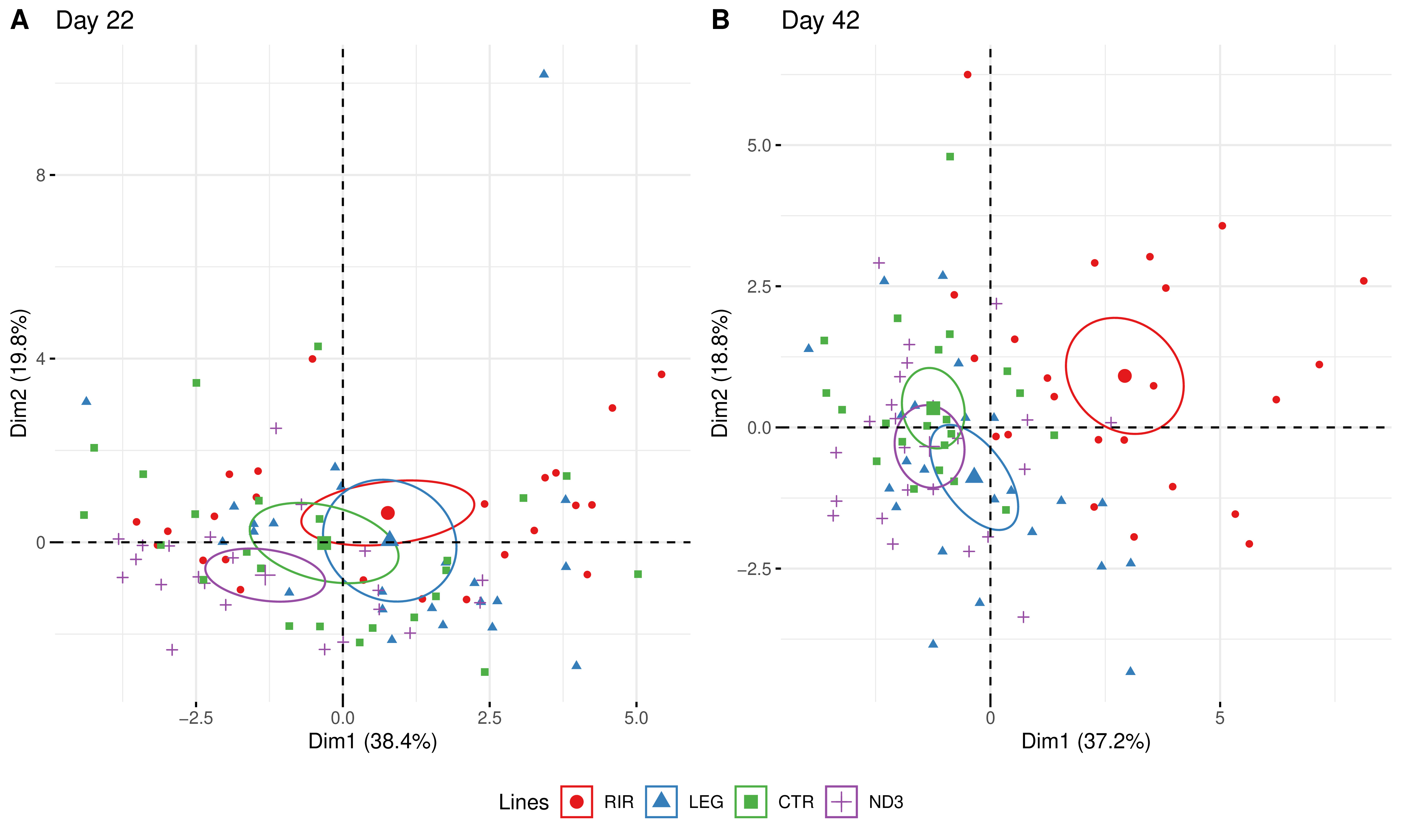

Supplement: Supplementary file 2 [file mmc2.jpg]
